# Investigation of the Relationship between Cerebral Near-Infrared Spectroscopy Measurements and Cerebrovascular Event in Coronary Artery Bypass Grafting Operation in Patients without Carotid Stenosis and Patients with Carotid Stenosis below Surgical Margins

**DOI:** 10.21470/1678-9741-2019-0050

**Published:** 2020

**Authors:** Ceyhun Coskun, Ferhat Borulu, Izzet Emir, Muhammed Hanedan, Ilker Mataraci

**Affiliations:** 1 Yalvac Public Hospital, Isparta, Turkey.; 2 Atatürk University, Medical Faculty, Cardiovascular Surgery Department, Erzurum, Turkey.; 3 Erzincan University, Medical Faculty, Cardiovascular Surgery Department, Erzincan, Turkey.; 4 Ahi Evren Thoracic Cardiovascular Surgery Education and Research Hospital, Trabzon, Turkey.

**Keywords:** Carotid Stenosis, Cardiopulmonary Bypass, Cerebrovascular Circulation, Arterial Pressure, Coronary Artery Bypass, Anesthesia, Patient Discharge

## Abstract

**Objectives:**

Stroke is an important cause of mortality and morbidity in surgery. In the present study, we examined the cerebral oximetry values of patients with carotid artery stenosis who did not present surgical indications and those who did not present carotid artery stenosis in coronary artery bypass grafting (CABG) surgery by comparing their cerebral oximetry values with cerebrovascular disease (CVD).

**Methods:**

Between January and May 2014, 40 patients who underwent isolated CABG were included in the study. Cerebral oximetry probes were placed prior to induction of anesthesia. Cerebral oximetry values were recorded before induction, in the pump (cardiopulmonary bypass) inlet period, in the post-clamp period, in the pump outlet period, and in the intensive care unit and neurological complications.

**Results:**

There was no difference between the groups in terms of demographic data and routine follow-up parameters. Intraoperative surgical data and early postoperative results were similar in both groups. When comparing the groups, there were no statistically significant results in cerebral oximetry values and CVD development. Only one patient in group 2 had postoperative CVD and this patient was discharged from the hospital with right hemiplegia. Mean arterial pressure (MAP)levels were significantly higher in Group 2 (*P*<0.05).

**Conclusion:**

The follow-up of cerebral perfusion with a method like near-infrared spectroscopy (NIRS) will ensure that MAP is adjusted with interventions that will be made according to changes in NIRS. Thus, it will be possible to avoid unnecessary medication and flow-rate increase with cerebral oxygen saturation (rSO_2_) follow-up.

**Table:** 

Abbreviations, acronyms & symbols				
ABP	= Arterial blood pressure		Hct	= Hematocrit
BD	= Base deficit		ICU	= Intensive care unit
CABG	= Coronary arterial bypass grafting		CO	= Cardiac output
CBF	= Cerebral blood flow		MAP	= Mean arterial pressure
COPD	= Chronic obstructive pulmonary disease		MRI	= Magnetic resonance imaging
CPB	= Cardiopulmonary bypass		NIRS	= Near-infrared spectroscopy
CPP	= Cerebral perfusion pressure		NSE	= Neuron-specific enolase
CVD	= Cerebrovascular disease		PaO_2_	= Partial pressure of arterial oxygen
CVE	= Cerebrovascular event		rSO_2_	= Cerebral oxygen saturation
ECG	= Electrocardiography		SEPs	= Somatosensory evoked potentials
EEG	= Electroencephalography		SpO_2_	= Peripheral oxygen saturation by pulse oximetry
ETCO_2_	= End-tidal carbon dioxide measurement		TCD	= Transcranial Doppler ultrasonography

## INTRODUCTION

Coronary artery disease, which is the narrowing or blockage of coronary arteries usually caused by atherosclerosis, is a major cause of death worldwide^[1]^. Coronary artery bypass grafting (CABG) is one of the approaches for treating coronary artery disease. Although CABG surgery has a mortality rate of <2%, there is a significant morbidity rate affecting the central nervous system^[2]^. Cerebrovascular accident(stroke) is one of the most important causes of morbidity after CABG surgery, and its incidence ranges from 1.3 to 4.3%^[3]^. Embolic debris from the heart and large vessels are the most important etiological factor for stroke^[4]^. In a previous study, carotid artery lesions were detected as one of the most important etiological factors for stroke after CABG surgery^[5]^. These factors should be investigated in the preoperative period, especially in high-risk patients, and the operation should be planned accordingly. Actions such as providing high perfusion pressure and avoiding intraoperative hypotension are important, particularly in hypertensive patients^[6]^. Apparently, accurate and effective monitoring during surgery reduces mortality and morbidity associated with CABG. Cerebral perfusion can be indirectly evaluated by mean arterial pressure (MAP), pulse and blood gas monitoring and by specific methods such as near-infrared spectroscopy (NIRS), electroencephalography (EEG), somatosensory evoked potentials (SEPs), transcranial Doppler ultrasonography (TCD), and monitoring of jugular venous oxygen saturation. Moreover, cerebral ischemia markers (lactate, S-100 protein, neuron-specific enolase [NSE]) are used in combination with the other evaluation methods.

None of the traditional methods can show cerebral perfusion instantaneously and locally during CABG surgery. The present study aims to determine if cerebral perfusion can be monitored instantaneously and locally using NIRS, which is a noninvasive and easy-to-use technique. Although many studies have used NIRS, this is the first to demonstrate the application of NIRS in adult patients with carotid stenosis undergoing cardiovascular surgery.

## METHODS

### Ethical Consideration

The study protocol was approved by the Ethics Committee of the Trabzon Kanuni Training and Research Hospital. All patients were informed about the study and all signed the informed consent form.

### Patient Selection

This study was planned by randomly selecting and prospectively examining 20 patients without carotid lesion and 20 patients with carotid lesions who had no surgical indication. All patients in the study were scheduled to undergo an elective CABG surgery at the Ahi Evren Thoracic and Cardiovascular Surgery Training and Research Hospital between January 2, 2014 and May 30, 2014. Patients who previously had a persistent cerebrovascular event were not included in the study.

### Study Design

Patients were divided into two groups according to the presence of carotid lesions: group 1 had 20 patients without carotid lesions and group 2 had 20 patients with carotid lesions without surgical indication. Preoperative Doppler ultrasound was performed for all patients. Magnetic resonance imaging (MRI) was performed when necessary. Lesions were detected with imaging tests. The study protocol was approved by the Ethics Committee of Trabzon Kanuni Training and Research Hospital. Patients were informed about the procedure, and the consent forms were obtained. Demographic characteristics (age and gender), clinical findings, presence of carotid artery stenosis, presence of chronic disease (diabetes mellitus, hypertension, chronic obstructive pulmonary disease, hyperlipidemia and chronic renal failure), smoking status, history of cerebrovascular event (CVE) and presence of peripheral arterial disease were collected from all patients.

After the patients were taken to the operating room, cerebral oxygen saturation (rSO_2_) probes were placed prior to induction of anesthesia. Cerebral oximetry values were recorded before the induction, in the pump inlet period, in the post-clamp period, in the pump outlet period, and in the intensive care unit (ICU).

All patients underwent elective CABG with the same surgical method under general anesthesia. Left internal mammary artery and saphenous vein grafts were prepared considering the number of vessels to be operated and the grafts to be used. Coronary bypass was performed using a standard cardiopulmonary pump.

Mechanical ventilation duration, intensive care and hospital stay, in addition to postoperative complications, were recorded.

### Statistical Consideration

Data were analyzed in SPSS software version 17.0. Continuous data were shown as arithmetic means ± standard deviation. Categorical data were expressed as number (%). The Student’s t-test was used for continuous data analysis and the chi-square test was used for categorical data analysis. Fisher’s exact test was used for some analyses, if necessary. Statistical significance was considered as *P*≤0.05.

## RESULTS

Forty random patients who underwent isolated elective CABG between January 2014 and May 2014 were included in the study. Of these, 20 without carotid lesion were placed in group 1 and 20 patients with carotid lesions without surgical indication were placed in group 2. The clinical characteristics of the two groups are summarized in Table 1; as shown, there was no significant difference in clinical characteristics between the two groups. Both groups consisted of patients who did not have a permanent cerebrovascular event prior to the operation. Only one patient in group 2 had a short-term history of hemiparesis nearly 5 years ago, without neurological deficits.

**Table 1:** Clinical characteristicsof patients.

		Group 1	Group 2	*P*-value
Age (years)		58±11	62.6±9.5	0.018
Size (cm)		170.75±9.1	165.6±10	0.099
Weight (kg)		80.85±12.44	75.15±8.98	0.106
Gender	Female, n (%)	3 (15%)	5 (25%)	
	Male, n (%)	17 (85%)	15 (75%)	0.69
COPD, n (%)		2 (10%)	5 (25%)	0.212
CRF, n (%)		1 (5%)	0 (0%)	0.311
Hypertension, n (%)		10 (50%)	15 (75%)	0.102
Hyperlipidemia, n (%)		6 (30%)	9 (45%)	0.327
Diabetes mellitus, n (%)		5 (25%)	14 (70%)	0.004
Smoke, n (%)		8 (40%)	9 (45%)	0.749
Old CVE, n (%)		0 (0%)	5 (25%)	0.047
PAD, n (%)		0 (0%)	3 (15%)	0.072

COPD=chronic obstructive pulmonary disease; CRF=chronic renal failure; CVE=cerebrovascular PAD=peripheral arterial disease

The distribution of patients according to carotid lesions is shown in Table 2.

**Table 2:** Distribution of patients according to carotid lesions.

Right	Mildstenosis	5 (25%)
	Moderatestenosis	5 (25%)
Left	Mildstenosis	2 (10%)
	Moderatestenosis	3 (15%)
Bilateral stenosis		5 (25%)

While postoperative cerebrovascular disease (CVD) was not seen in group 1, 1 (5%) patient of group 2 had postoperative CVD and this patient was discharged from the hospital with right hemiplegia. This patient, included in group 2, had moderate stenosis in preoperative carotid artery ultrasonography. This patient was 57 years old and had no risk factors, except for hypertension. When the rSO_2_ values of this patient were analyzed, it was lower than the rSO_2_ values of the other patients in their group but showed no significant difference.

There was no significant difference between the intraoperative surgical data and the early postoperative results (Table 3).

**Table 3:** Intraoperative and postoperative early period findings.

	Group 1	Group 2	*P*-value
Pump time (min.)	87.25	81.85	0.550
Cross-clamp time(min.)	57.70	59.20	0.801
Intubation time (hours)	9.41	23.33	0.234
Lengthof intensive care (days)	3.25	3.85	0.407
Hospital stay (days)	7.95	9.50	0.185

In our study, rSO_2_ values obtained prior to anesthetic induction (normal body temperature, 36-37°C, and stable hemodynamic status) decreased during cooling (34ºC and 32ºC) in group 1 but increased in group 2. We found that rSO_2_ values started increasing with rewarming (34°C) in both groups, and rSO_2_ values decreased in both groups at the end of warming and when the patients were transferred to the ICU. When all patients were evaluated, no significant differences were found between the measurements before induction of anesthesia and other measurements (*P*>0.05). There was no significant difference in these changes when the groups were compared (*P*>0.05). In our study, there were no significant differences in terms of neurological event. However, when the MAP levels recorded in the measurement periods for rSO_2_ were evaluated, they were significantly higher in group 2 (*P*<0.05) than in group 1. Absence of significant difference in terms of neurological event could be provided with high MAP in group 2.

Furthermore, we recorded the arterial blood gas results of the patients in the periods when rSO_2_ was recorded (Table 4). Although there was no difference in partial alveolar carbon dioxide pressure (PaCO_2_) levels between the groups in each period, there was a statistically significant difference in PaCO_2_ levels between the pre-induction period, pump input, and pump exit periods (*P*<0.05). However, these changes were not correlated with changes in rSO_2_. There were no significant differences in arterial oxygen saturation (SaO_2_) levels between the groups, and differences in changes in SaO_2_ levels between the different measurement time points (*P*>0.05). At the same time, there was no correlation between changes in SaO_2_ levels and rSO_2_ levels. To evaluate the total body perfusion status of patients during cerebral perfusion evaluation, we recorded pH, HCO^-^_3_, and base deficit (BD) values as indirect indicators of total body perfusion at the same time points. The α-stat method was applied in our clinic as a control of acid-base balance, and bicarbonate infusion was performed in case of increased BD accompanying acidosis. There was no significant difference between the groups in terms of pH levels and changes in the measurement time points (*P*>0.05). HCO^-^_3_ levels between groups at all measurement levels, except in the pre-induction period, showed a statistically significant difference (*P*<0.05). There was no statistically significant difference in BD levels between the pre-induction period and the other measurement time points (*P*>0.05). Measurements at the pump inlet, cross-clamp, and pump outlet periods significantly differed between the groups (*P*<0.05). When these changes were evaluated with change in the rSO_2_ level, no correlation was found.

**Table 4:** Cerebral oxymetry analysis between groups during different periods.

		Pre-induction		Pump start		After cross-clamping		Pump end		Intensive care unit	
			*P*		*P*		*P*		*P*		*P*
CO(right)(%)	Group 1	64.15	0.321	64.7	0.235	62.35	1	62.65	0.293	59.85	0.984
	Group 2	61.85		61.4		62.35		64.85		59.9	
CO (left)(%)	Group 1	64.65	0.252	66.7	0.131	63.3	0.577	65	0.529	60.75	0.676
	Group 2	61.45		61.05		61.55		63.3		59.65	
Peripheral O_2_	Group 1	97.05	0.578	97.95	0.394	98	0.511	98.05	0.197	98.55	0.399
	Group 2	97.7		97.1		98.45		98.8		97.8	
MAP (mmHg)	Group 1	93.8	0.008	65.1	0.609	57.85	0.001	64.05	0.245	77.95	0.111
	Group 2	78.1		67.1		75.85		70.45		70.35	
Temperature (ºC)	Group 1	36.28	0.903	33.83	0.278	32.27	0.687	36.8	0.626	36.79	0.006
	Group 2	36.26		34.42		32.5		36.65		36.49	
pH	Group 1	7.42	0.51	7.41	0.941	7.42	0.119	7.36	0.199	7.36	0.012
	Group 2	7.43		7.41		7.39		7.32		7.31	
pCO_2_ (mmHg)	Group 1	35.16	0.015	37.53	0.023	36.3	0.194	40.88	0.008	41.87	0.118
	Group 2	30.39		32.02		33.66		34.69		38.03	
pO_2_ (mmHg)	Group 1	106.44	0.087	258.7	0.385	249.2	0.045	175.24	0.605	162.9	0.547
	Group 2	138.85		234.85		291.25		192.3		175.32	
HCO^-^_3_ (mmol/L)	Group 1	23.98	0.202	24.13	0.002	23.94	0.003	22.46	0.001	23.25	0.001
	Group 2	23.05		21.23		21.52		19.08		19.67	
BE (mmol/L)	Group 1	-0.89	0.138	-0.8	0.002	-0.49	0.001	-2.01	0.199	-1.24	0.001
	Group 2	-2.27		-4.1		-3.81		-6.55		-6.54	
Hematocrit (%)	Group 1	40.31	0.117	29.24	0.781	27.4	0.325	29.25	0.067	31.12	0.233
	Group 2	37.31		29.69		26.33		26.85		29.65	
Glucose (mg/dL)	Group 1	133.4	0.299	141.25	0.028	153	0.038	198.95	0.833	196.1	0.272
	Group 2	151.5		176.6		202.95		202.95		217.55	

MAP=mean arterial pressure; CO=cerebral oximetry

## DISCUSSION

One of the main goals during anesthesia applications is to provide adequate tissue oxygenation, and different monitoring parameters are used for this purpose. The methods used during conventional anesthesia are electrocardiography (ECG), noninvasive arterial blood pressure (ABP) measurement, and peripheral oxygen saturation by pulse oximetry (SpO_2_). More advanced monitoring systems include end-tidal carbon dioxide measurement (ETCO_2_), monitoring of airway pressure changes, invasive ABP, central venous pressure, pulmonary artery occlusion pressure, mixed venous oxygen saturation monitoring, blood gas analysis, and urinary output rate follow-up.

To minimize the effects of cardiopulmonary bypass (CPB) during open heart surgery, it is not always appropriate to maintain tissue and organ homeostasis of close to the physiological limits. In this case, it is important to determine and follow the confidence intervals for tissues and organs based on instant conditions. The brain is one of the most affected organs during CPB. Due to its low tolerance to hypoxia and the dramatic clinical results in case of brain damage, brain perfusion follow-up and protection studies are gaining importance^[7,8]^. Several methods have been used to monitor adequate perfusion to the brain and to evaluate its metabolic activity. Cerebral perfusion is indirectly evaluated using MAP or pulse and blood gas monitoring, NIRS, electroencephalography (EEG), and SEPs. It can also be measured using specific methods such as jugular venous oxygen saturation monitoring and cerebral ischemia markers (such as lactate, S-100 protein, and neuron-specific enolase [NSE])^[9,10]^. The effects of parameters such as MAP, SaO_2_, PaCO_2_, acid-base balance (pH), hematocrit (Hct) and body temperature on cerebral perfusion during CPB have been shown in several studies^[7,11]^. In a study on pigs by Ehrlich et al.^[12]^, cerebral blood flow (CBF) and cerebral metabolic rate measured at 37°C (baseline value) decreased in mild (28°C) and deep (18°C) hypothermia and increased in very deep (8°C) hypothermia. It is generally assumed that cerebral metabolism stops completely at 18°C. In the same study, the authors asserted that the brain still had basal metabolism at 18°C, and that cerebral protection increased but was not complete. They suggested further cooling in order to stop cerebral metabolism.

In a study by Sungurtekin et al.^[13]^, it was suggested that MAP, rather than the pump flow rate, was effective in cerebral perfusion, and that pump flow was not effective in cerebral perfusion when considered independent of MAP. In a study conducted by Tufo et al.^[14]^, the authors compared the neurological outcome parameters of patients with a MAP of <40 mmHg and those with a MAP of >60 mmHg during CPB, and found that patients with MAP values of <40 mmHg had three times more neurological problems than patients with MAP >60 mmHg. In our study, there was no significant difference in terms of neurological events. However, when MAP levels were evaluated at the measurement time points for rSO_2_, they were significantly higher in group 2 (*P*<0.05). No significant difference in neurological event could be provided with high MAP in group 2.

In a previous study by Gersten, cerebral perfusion pressure (CPP), partial pressure of arterial oxygen (PaO_2_), cerebral metabolism, partial pressure of arterial carbon dioxide (PaCO_2_), and cardiac output (CO) were identified as the main factors in the control of cerebral blood flow. In that study, it was reported that PaCO_2_ increases cerebral blood flow independent of its autoregulatory mechanism, and this increase occurs by cerebral vascular dilatation. In our study, we recorded the arterial blood gases of patients in periods in which cerebral oxygen saturation (rSO_2_) was recorded. Although there was no difference between the groups at each time point, the difference in the PaCO_2_ level among the pre-induction period, pump input, and pump exit time points were significantly different (*P*<0.05). However, these changes were not correlated with changes in the rSO_2_ level. There was no significant difference in levels and changes of arterial oxygen saturation (SaO_2_) between the groups at the different measurement time points (*P*>0.05). No correlation was observed between changes in SaO_2_ and rSO_2_.

Luz et al. compared acid-base balances in patients at normothermia (37°C) and mild/moderate hypothermia (35-33°C). They then evaluated parameters such as pH, arterial bicarbonate and BDs, and no statistically significant differences was found in these parameters^[15]^. Similarly, no significant differences in blood gas values were observed in our study.

No significant difference was found between cross-clamp time and rSO_2_ values when both groups were compared (*P*>0.05).

### Study Limitations

-Neurological complications that developed in patients were evaluated as major neurological complications, and there was no comparison between minor neurological complications.
-The sample size was small, and the observational period was short, both of which could limit significant relationships to be obtained from the data. Future large-scale, multicenter studies with a longer follow-up duration are required.

## CONCLUSION

Stroke is an important cause of morbidity and mortality in patients undergoing CABG. Cerebral embolism or cerebral hypoperfusion is the most important cause of stroke in open heart surgery. We believe that instant perfusion monitoring is important for early detection of cerebral hypoperfusion or to avoid unnecessary high flow rates. We believe that NIRS, which is a noninvasive and easy-to-use technique to instantly evaluate cerebral perfusion, will be useful for this purpose. MAP can be adjusted with close monitoring of cerebral perfusion using a method such as NIRS, particularly in patients with carotid artery stenosis, and technical and medical interventions can be carried out according to the change in NIRS. Thus, unnecessary increases in the perfusion flow rate and unnecessary use of medication can be avoided by rSO_2_ monitoring and ensuring that it stays within the safe margin.

**Table:** 

Authors' roles & responsibilities	
CC	Substantial contributions to the conception or design of the work; or the acquisition, analysis, or interpretation of data for the work; drafting the work or revising it critically for important intellectual content; final approval of the version to be published
FB	Drafting the work or revising it critically for important intellectual content; final approval of the version to be published
IE	Agreement to be accountable for all aspects of the work in ensuring that questions related to the accuracy or integrity of any part of the work are appropriately investigated and resolved; final approval of the version to be published
MH	Drafting the work or revising it critically for important intellectual content; final approval of the version to be published
IM	Final approval of the version to be published
